# Epigenomics of being bullied: changes in DNA methylation following bullying exposure

**DOI:** 10.1080/15592294.2020.1719303

**Published:** 2020-01-28

**Authors:** Rosa H. Mulder, Esther Walton, Alexander Neumann, Lotte C. Houtepen, Janine F. Felix, Marian J. Bakermans-Kranenburg, Matthew Suderman, Henning Tiemeier, Marinus H. van IJzendoorn, Caroline L. Relton, Charlotte A. M. Cecil

**Affiliations:** a Institute of Education and Child Studies, Leiden University, Leiden, The Netherlands; b Department of Child and Adolescent Psychiatry/Psychology, Erasmus MC, University Medical Center Rotterdam, Rotterdam, The Netherlands; c Generation R Study Group, Erasmus MC, University Medical Center Rotterdam, Rotterdam, The Netherlands; d MRC Integrative Epidemiology Unit, Population Health Sciences, Bristol Medical School, University of Bristol, Bristol, UK; e Department of Psychology, University of Bath, Bath, UK; f Lady Davis Institute for Medical Research, Jewish General Hospital, Montreal, Qc, Canada; g Department of Pediatrics, Erasmus MC, University Medical Center Rotterdam, Rotterdam, The Netherlands; h Department of Epidemiology, Erasmus MC, University Medical Center Rotterdam, Rotterdam, The Netherlands; i Clinical Child & Family Studies, Vrije Universiteit Amsterdam, Amsterdam, The Netherlands; j Department of Social and Behavioral Science, Harvard TH Chan School of Public Health, Boston, MA, USA; k Department of Psychology, Education and Child Studies, Erasmus University Rotterdam, Rotterdam, The Netherlands; l School of Clinical Medicine, University of Cambridge, Cambridge, UK; m Department of Psychology, Institute of Psychology, Psychiatry & Neuroscience, King’s College London, London, UK

**Keywords:** EWAS, bullying, DNA methylation, longitudinal, ALSPAC, Generation R, peer victimization, Illumina 450K

## Abstract

Bullying among children is ubiquitous and associated with pervasive mental health problems. However, little is known about the biological pathways that change after exposure to bullying. Epigenome-wide changes in DNA methylation in peripheral blood were studied from pre- to post measurement of bullying exposure, in a longitudinal study of the population-based Generation R Study and Avon Longitudinal Study of Parents and Children (combined *n *= 1,352). Linear mixed-model results were meta-analysed to estimate how DNA methylation changed as a function of exposure to bullying. Sensitivity analyses including co-occurring child characteristics and risks were performed, as well as a Gene Ontology analysis. A candidate follow-up was employed for CpG (cytosine-phosphate-guanine) sites annotated to *5-HTT* and *NR3C1*. One site, cg17312179, showed small changes in DNA methylation associated to bullying exposure (*b *= −2.67e-03, SE = 4.97e-04, *p *= 7.17e-08). This site is annotated to *RAB14*, an oncogene related to Golgi apparatus functioning, and its methylation levels decreased for exposed but increased for non-exposed. This result was consistent across sensitivity analyses. Enriched Gene Ontology pathways for differentially methylated sites included cardiac function and neurodevelopmental processes. Top CpG sites tended to have overall low levels of DNA methylation, decreasing in exposed, increasing in non-exposed individuals. There were no gene-wide corrected findings for *5-HTT* and *NR3C1*. This is the first study to identify changes in DNA methylation associated with bullying exposure at the epigenome-wide significance level. Consistent with other population-based studies, we do not find evidence for strong associations between bullying exposure and DNA methylation.

## Introduction

The social environment is a major contributor to mental health. Bullying is a ubiquitous social stressor, with worldwide estimates ranging from one in ten to almost half of all children that are exposed[1]. Following Olweus’ definition, a person is being bullied ‘when he or she is exposed, repeatedly and over time, to negative actions on the part of one or more other persons’. Such negative actions should be intentional and performed by someone perceived be more powerful than the subject. Actions can include physical behaviours, such as hitting and kicking, verbal behaviours, such as calling names, as well as indirect or relational behaviours, such as social exclusion [2]. Bullying exposure (i.e. bullying victimization) has been associated with numerous mental health issues including behavioural problems, depressive symptoms [3–6] and suicidal ideation [7]. However, whereas a myriad of harmful and persistent psychiatric consequences of being bullied have been identified, the biological pathways that change after exposure to bullying remain largely uncharted. Identifying these pathways is a pivotal step in understanding how peer-inflicted stress affects the human body.

Research on other environmental stressors, such as parental abuse [8], prenatal maternal stress [9,10] or childhood trauma in general [11–15] has incorporated epigenetic data to investigate the hypothesis that stressors affect the molecular configuration on and around the DNA, thereby influencing its functionality, with potential downstream effects on stress reactivity and mental health [16–19]. One often studied epigenetic mechanism is DNA methylation, in which a methyl-group binds to a cytosine nucleotide of the DNA (cytosine-phosphate-guanine site or CpG site). Whereas early epigenetic studies focused on DNA methylation of a single candidate gene, there has been an increase in hypothesis-free epigenome-wide methylation studies (EWASs) investigating DNA methylation levels of hundreds of thousands of CpG sites (CpGs) across the genome. One study [11], for example, found multiple epigenome-wide significant differentially methylated CpGs related to different types of childhood maltreatment.

In contrast to other forms of adversity, research on bullying exposure and epigenetics is markedly scarce. To the best of our knowledge, only three such studies have been performed. In 28 monozygotic twin pairs discordant for bullying exposure [20], increased levels of methylation were observed in the serotonin transporter gene (*5-HTT)* promoter region for the exposed twin siblings from 5 to 10 years, but not for the non-exposed twin siblings. Another study in 1,149 13 to 14 year old children found bullying exposure to be associated with increased methylation levels of exon 1F of the glucocorticoid receptor gene (*NR3C1*) [21]. Further, an EWAS was performed on bullying exposure in 1,658 twins [13], thereby expanding the search for differentially methylated sites beyond the ‘usual suspects’, i.e. candidate genes that have been firmly implicated in neurotransmitter and hormonal functions, to enable the identification of potentially novel biological pathways. Bullying exposure during childhood as reported by mother and child at age 7–12 years, and bullying exposure during adolescence, retrospectively reported by the child at 18 years, were however not related to differential methylation. Given that DNA methylation is expected to change over time [22] due to both extrinsic as well as intrinsic factors, a model in which DNA methylation both before and after bullying exposure is taken into account should be more sensitive to the effects of exposure.

In the current study, we made use of two population-based cohorts featuring repeated measures of DNA methylation to characterize longitudinal epigenome-wide associations with bullying exposure. Longitudinal mixed models were performed separately in the two cohorts to identify associations between exposure to bullying and changes in DNA methylation from pre- to post bullying report. Results were then meta-analysed to maximise statistical power and to evaluate coherence among the estimates derived from the two populations. Epigenome-wide associations with bullying were studied in a hypothesis-free analysis. In a secondary candidate gene follow-up analysis we examined DNA methylation at *5-HTT* and *NR3C1* for gene-wide associations with bullying.

## Results

### Sample characteristics

Sample characteristics are described in Table 1. In Generation R, bullying exposure was reported by the mother at the mean (SD) age of 8.1 (0.1) years, and DNA methylation was measured at 6.0 (0.3) years and 9.8 (0.3) years of age. In the Avon Longitudinal Study of Parents and Children (ALSPAC), bullying exposure was reported by the child at the mean (SD) age of 8.6 (0.2) years, and DNA methylation was measured at 7.5 (0.1) and 17.1 (1.0) years of age (Supplemental Figure 1). In the main analysis 45.5% of children in the Generation R sample and 39.4% of children in the ALSPAC sample were categorized as exposed to bullying victimization. In the sensitivity analysis with a more stringent definition of bullying, these numbers are 9.9% and 12.1%, respectively. The current selected samples for each cohort were compared with (i) a set of participants with complete data on covariates, and (ii) a set of participants with complete data on both covariates and bullying exposure (full details in Supplemental Table 1). This showed that children in the current sets had a higher gestational age and higher SES, had mothers who were older, had a higher non-verbal intelligence quotient (IQ) score and were older at bullying exposure report. In Generation R, but not ALSPAC, children in the selected sample also had a lower Body Mass Index (BMI), less behavioural problems prior to exposure, and had less reported stressful experiences other than bullying exposure. No differences were found for child sex, or bullying exposure.

**Table 1.** Sample characteristics.

	Generation R (*n =* 506)		ALSPAC (*n =* 846)	
Age in years bullying exposure report (mean (SD))	8.1 (0.1)		8.6 (0.2)	
Sex (No. (%) boys)	251 (49.6)		407 (48.3)	
Gestational age in weeks (mean (SD))	40.2 (1.4)		39.6 (1.5)	
Maternal education (No. (%))				
Low	21 (4.2)		72 (8.5)	
Medium	101 (20.0)		336 (39.7)	
High	384 (75.9)		438 (51.8)	
Maternal age at delivery (mean (SD))	32.8 (3.9)		29.7 (4.4)	
Bullying exposure (No. (%) yes)	229 (45.3)		333 (39.4)	
Bullying exposure - sensitivity analysis (No. (%) yes)	50 (9.9)		102 (12.1)	
Behavioural problem score (mean (SD)) (GenR *n*=451, ALSPAC *n*=794)	17.3 (12.2)		6.9 (3.9)	
Intelligence quotient (mean (SD)) (GenR *n*=465, ALSPAC *n*=811)	107.3 (14.0)		102.6 (16.7)	
Other stressful experiences (mean (SD)) (GenR *n*=482, ALSPAC *n*=597)	3.7 (2.1)		1.5 (1.4)	
Alcohol use (mean (SD)) (ALSPAC *n*=624)			8.1 (4.8)	
Methylation measurement	T1 (*n* = 404)	T2 (*n* = 391)	T1 (*n* = 820)	T2 (*n* = 819)
Age in years DNA methylation (mean (SD))	6.0 (0.3)	9.8 (0.3)	7.5 (0.1)	17.1 (1.0)
BMI in kg/m^2^ (mean (SD))	15.9 (1.3	17.1 (2.0)	16.2 (2.0)	22.6 (3.6)
*AHRR* CpG quintiles (No. (%))				
0.769, 0.873	93 (23.0)	66 (16.9)	123 (15.0)	205 (25.0)
0.873, 0.891	79 (19.6)	80 (20.5)	146 (17.8)	182 (22.2)
0.891, 0.906	72 (17.8)	87 (22.3)	171 (20.9)	157 (19.2)
0.906, 0.920	73 (18.1)	86 (22.0)	186 (22.7)	142 (17.3)
0.920, 0.963	87 (21.5)	72 (18.4)	194 (23.7)	133 (16.2)

SD: standard deviation; No.: number; T1: time point 1; T2: time point 2; BMI: Body Mass Index

### Comparison of longitudinal epigenome-wide association studies

The separate longitudinal EWASs for the two cohorts (Q-Q plots in Supplemental Figure 3), identified no epigenome-wide Bonferroni-significant associations, with a lowest obtained *p*-value of *p *= 5.93x10^−06^ (CpG site cg034529555, annotated to *NAV2*) for Generation R and of *p *= 1.08x10^−06^ (CpG site cg24506221, annotated to *GSTM1*) in ALSPAC. Estimates for bullying exposure among the top 1000 CpG sites in each cohort more often had a negative direction (79.8% in Generation R, 66.3% in ALSAPC) than would be expected by chance (*Χ^2^*(1) = 355.22, *p *< 2.20x10^−16^ for Generation R, *Χ^2^*(1) = 106.28, *p *< 2.20x10^−16^ for ALSPAC). Bullying exposure estimates among the top 1000 in Generation R were not significantly correlated with those in ALSPAC (*r*(998) = −0.05, *p *= 1.09x10^−01^). The correlation between the top 1000 in ALSPAC was slight but significantly negative (*r*(998) = −0.07, *p *= 1.09x10^−02^) with those in Generation R.

### Meta-analysis

**Figure 1.**
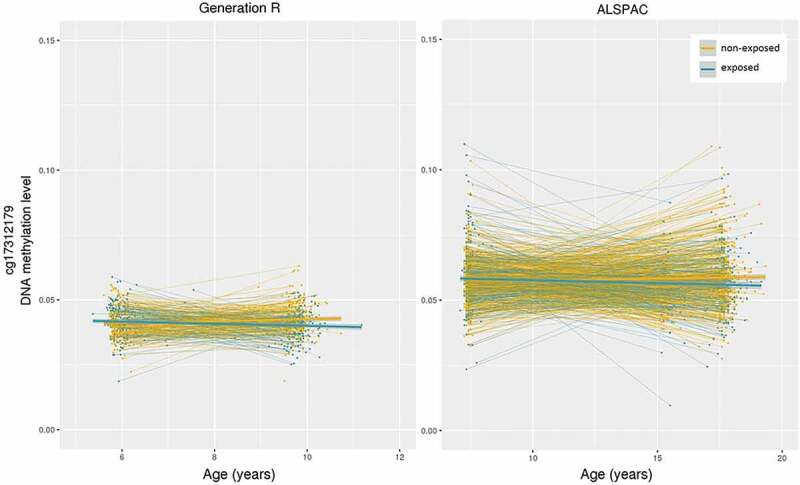
Change in DNA methylation pre- and post- bullying exposure measurement for exposed and non-exposed in Generation R and ALSPAC. Data are residualized for covariates present in linear mixed model.

In the meta-analysis, one CpG site was significantly associated with bullying exposure: cg17312179 (*b *= −2.67x10^−03^, SE = 4.97x10^−04^, *p *= 7.17x10^−08^; Supplemental Figure 4), a site in the leader sequence (5'UTR) of the *RAB14* gene, located on chromosome 9. In Generation R (*b *= −2.47x10^−03^, SE = 6.17x10^−04^, *p *= 6.25x10^−05^), DNA methylation of this CpG site increased on average 0.13% from the mean age of 6.0 to 9.8 years in non-exposed, but decreased −0.12% in exposed. In ALSPAC (*b *= −3.05x10^−03^, SE = 8.36x10^−04^, *p *= 2.64x10^−04^) methylation of this CpG site increased 0.09% from the mean age of 7.5 to 17.1 years in non-exposed, whereas it decreased −0.21% in exposed (Figure 1). DNA methylation differences at cg17312179 between the non-exposed and the exposed group were not present before exposure measurement, but were so after (Supplemental Analysis). The ten CpGs with the lowest *p*-values are shown in Table 2 (see Supplemental Table 2 for associated functions).

**Table 2.** Ten CpG sites with lowest *p*-values in meta-analysis of epigenome-wide associations with bullying exposure.

CpG site	Gene	Chr	Relation to gene	Relation to CpG site	*B* (SE)	*p*-value	Direction of change exposed/non-exposed
cg17312179	*RAB14*	9	5'UTR	Island	−2.67e-03 (4.97e-04)	7.17e-08	-/+
cg09291817	*MAZ*	16	TSS1500	Island	−2.01e-03 (4.03e-04)	6.21e-07	-/+
cg11278602	*HCG4*	6	Body	Island	−2.98e-03 (6.41e-04)	3.35e-06	-/+
cg00911813	*TNRC18*	7	5'UTR	Island	−1.32e-03 (2.91e-04)	5.37e-06	-/+
cg08971637	*DGUOK*	2	TSS1500	N shore	−9.63e-03 (2.14e-03)	6.66e-06	-/+
cg12767834	*SNPH*	20	5'UTR	Island	−2.01e-03 (4.46e-04)	7.01e-06	-/+
cg26394220	*MIR375;* *CCDC108*	2	TSS1500; Body	Island	−2.40e-03 (5.40e-04)	8.92e-06	-/+
cg19790568	*PRX*	19	Body	Island	−1.49e-03 (3.36e-04)	9.08e-06	-/+
cg10929442	*ST8SIA4*	5	Body	N shore	−1.01e-03 (2.29e-04)	9.60e-06	-/+
ch.4.134822993R		4			−1.20e-03 (2.72e-04)	1.11e-05	-/+

SE: standard error; exposed/non-exposed; exposed to bullying victimization/not exposed to bullying victimization

The top 1000 CpGs from the meta-analysis had a higher representation of CpGs with a negative estimate for bullying exposure (83.5%) than all other CpGs (58.2%, *Χ^2^*(1) = 261.07, *p *< 2.20x10^−16^). Moreover, the top 1000 CpGs had a higher representation of CpGs with a positive age estimate (representing change in non-exposed, 79.2%) than the other CpGs (65.1%, *Χ^2^*(1) = 87.29, *p *< 2.20x10^−16^), and a higher representation of CpGs with low levels of DNA methylation (mean β value<0.2 in both Generation R and ALSPAC, threshold as elsewhere [23]) at both time points (55.7% versus 37.0%, *Χ^2^*(1) = 149.63, *p *< 2.20x10^−16^), and more often located in promoters (28.8% versus 20.1%, *Χ^2^*(1) = 46.66, *p *= 8.45x10^−12^) and CpG islands (41.8% versus 30.8%, *Χ^2^*(1) = 56.63, *p *= 5.25x10^−14^). Together, this indicates that top sites tended to be located in promoters and CpG islands, and to have overall low levels of DNA methylation, decreasing in exposed, while increasing in non-exposed. See Table 3 for these characteristics at multiple *p*-thresholds.

**Table 3.** Characteristics of CpG sites selected for various levels of significance in meta-analysis.

CpG sites	*n*	Negative bullying exposure coefficient (%)	Positive coefficient non-exposed (%)	Low methylation (%)	Promoter associated (%)	CpG island associated (%)
all	473864	58.3	65.1	37.0	20.1	30.8
*P* < 0.1	53168	71.2**	69.2**	44.1**	23.1**	34.6**
*P* < 0.01	5997	78.6**	75.3**	50.5**	26.3**	37.9**
*P* < 0.001	644	85.1**	79.7**	56.4**	28.1**	40.8**
*P* < 0.0001	66	86.4**	83.3**	62.1**	30.3**	47.0**
*P* < 0.00001	9	100.0**	100.0*	88.9**	44.4	77.8*

CpG sites were classified as having low methylation if β value<0.2 in Generation R and ALSPAC.

* *p* <0.05 compared to all other CpG sites, ** *p*<0.001 compared to all other CpG sites

### Follow-up analyses

#### Sensitivity analyses

A series of sensitivity analyses were performed on cg17312179 in each cohort and then meta-analysed. First, an analysis was performed with a more stringent definition of bulling exposure. Second, we reran separate analyses or additionally adjusting for (i) BMI; (ii) pre-existing behaviour problems; (iii) non-verbal intelligence quotient (IQ); (iv) stressful experiences other than bullying exposure; and (v) alcohol use. The bullying exposure coefficients from sensitivity analyses were not different from the bullying exposure coefficient from the main analysis (lowest *p *= 7.42x10^−02^). ‘Other stressful experiences’ was the only added variable that independently associated with cg17312179 (*b *= 2.20x10^−04^, SE = 9.96x10^−05^, *p *= 2.73x10^−02^).

#### Genetic associations

A triad of look-ups did not show evidence of genetic associations with cg17312179 methylation. First, the probe was not present in a list of polymorphic probes [24]. Second, no *cis* or *trans* meQTLs were found to associate with this probe [25], and third, low additive genetic influences (1.79x10^−10^%) and high shared (34.9%) and non-shared (65.1%) environmental influences have been reported for this probe based on twin heritability analyses [26].

#### Look-up of previous findings in the literature

Results from eleven EWASs on childhood adversity [11,13,15,16,27–32] were searched for cg17312179 or other CpGs annotated to *RAB14*. No *RAB14*-associated probes were reported. Since cg17312179 was not reported in these studies, we could not establish if the direction of association was congruent with the one currently reported.

#### Candidate gene-wide analyses

Results from the meta-analysis were separately studied for probes annotated to *5-HTT* and *NR3C1* (Supplemental Figure 5). None of the probes reached gene-wide Bonferroni-significance (thresholds *p *= *3*.13x10^−03^ for *5-HTT* and *p *= 1.22x10^−03^ for *NR3C1*).

#### Functional associations

Gene Ontology (GO) analysis on CpGs with *p *< 0.001 (*n = *644 CpGs, *n *= 396 genes) yielded 126 pathways, 25 of which were confirmed by a GO analysis on CpGs with *p *< 0.01 (*n *= 5997 CpGs, *n *= 3722 genes) and 43 of which were confirmed by a GO analysis on CpGs with *p *< 0.0001 (*n *= 66 CpGs, *n *= 53 genes). Ryanodine-sensitive calcium-release channel activity as the most enriched (*p *= 9.99x10^−08^) biological process (Supplemental Tables 4–6, Supplemental Figure 6). Three isoforms of the ryanodine receptors exist [33], RYR1, RYR2, and RYR3, each present in a different tissue. Here, *RYR2* was part of the GO pathway, a gene specifically active in the heart tissue. Other enriched terms for biological processes involve various neurodevelopmental processes, such as astrocyte differentiation and action potential regulation, as well as processes such as muscle fibre development.

## Discussion

The current study is the first to characterize epigenome-wide intra-individual changes in DNA methylation related to bullying exposure. Our meta-analysis identified a CpG site with increasing levels of DNA methylation in non-exposed but decreasing levels in the exposed group. Other research [24–26] on this probe suggests that variance in DNA methylation at this CpG is primarily explained by environmental influences, with weak evidence of genetic effects. Sensitivity analyses showed that this association was not explained by co-occurring child characteristics, co-occurring risks, or consequences of bullying, including pre-existing behavioural problems, IQ, BMI, alcohol use or exposure to stressful experiences other than bullying. The site is located in the 5' untranslated region of *RAB14*, a member of the Ras oncogene family of GTPases. Ras GTPases are important in cellular signalling and RAB14 is involved in vesicle transport and Golgi apparatus functioning [34], and is expressed in multiple tissues (Supplemental Figure 7). No *RAB14*-associated probes have been reported in previous EWAS on childhood adversity.

*RAB14* expression has however been associated to stress in different tissues. In rat hippocampus it was shown to be downregulated after prenatal stress [35] and upregulated after mild chronic stress in stress-resilient rats [36], possibly marking an adaptive response. In humans, its expression was found to be reduced in the prostate of men with prostate cancer after nutrition and lifestyle intervention focused on stress reduction [37]. Also, *RAB14* expression in human brain tissue has been linked to depression and suicide [38], as was Syntaphilin, a protein that regulates synaptic vesicle processing [39] and encoded by *SYNPH*, a gene associated to one of the top 10 CpG sites from the meta-analysis. How the observed changes in *RAB14* methylation might relate to expression levels in this gene and what the downstream effects of these changes might be, however, remains to be elucidated in future functional studies.

GO analysis showed enrichment of the biological process of ryanodine-sensitive calcium release channel activity; these channels are a pathway important in cardiac functioning and the fight-or-flight response [40]. This finding is congruent with several other enriched pathways associated with cardiac functioning, and fits with GO findings from other research on epigenetics and physical abuse [11]. Further, GO analysis showed many neurodevelopmental processes, such as neuron differentiation, a biological pathway in which two of the associated genes, *SYNPH* and *ST8SIA4*, were among the top meta-analysis hits. ST8SIA4 (CMP-N-acetylneuraminate-poly-alpha-2,8-sialyltransferase) is present in the Golgi apparatus, involved in neural plasticity [41], and *ST8SIA4* knockout mice have been shown to display a decreased motivation for social interaction [42]. Functioning of both genes has been associated with brain disorders, such as schizophrenia [43,44] and Alzheimer’s disease [45,46]. Interestingly, in contrast to previous studies [20,21], no associations with bullying exposure were found at candidate genes *5-HTT* and *NR3C1*. Failure to replicate candidate epigenetic studies with epigenome-wide analyses is not uncommon [13]. This discrepancy may be explained by the stricter multiple testing correction applied in (candidate gene analyses as part of) epigenome-wide studies, or in the different specific regions tested by targeted gene approaches and microarray studies, rendering direct comparison unfeasible.

A pattern emerged of enrichment for CpGs with low overall methylation levels, increased over time, but decreased for exposed individuals. Furthermore, the top CpGs from the meta-analysis were more often located in CpG islands and promoter regions than would be expected by chance. Together this might indicate that bullying exposure is associated with an overall delayed downregulation of gene expression. However, promoter regions typically have low levels of methylation, and the enrichment of CpGs with low overall methylation levels in general seems to be more pronounced than the enrichment of promoter CpGs. In an EWAS on childhood abuse and promoter DNA methylation in adulthood [8], the stressor was also more often negatively than positively associated with DNA methylation. In another EWAS on childhood maltreatment and DNA methylation around 10 years of age (range 5–14 years) [23], researchers found an enrichment of CpGs with low methylation levels, often located in promoter regions as we did in the current study, but the association with maltreatment was more often positive. Unfortunately, direct comparison among studies is not straightforward because of timing differences in the measurement of DNA methylation, as well as the inherently unclear timing of often retrospectively reported stressors [32]. In the current study, effect estimates for the top ranking CpGs were incongruent and even seemed slightly oppositional between the two cohorts. One explanation might be the longer time period between bullying exposure and the DNA methylation measurement in ALSPAC. One study on timing differences in ALSPAC for example, found that recency of adversity exposure was more important in explaining DNA methylation levels than accumulation of adversity, regardless of timing [32]. On the other hand, even among top ranking CpGs associations were weak. Such effect sizes are in line however, with other epigenome-wide studies in population-based samples [13,15,47–49], where exposures are generally less extreme and abundant than in risk samples that typically encounter larger effect sizes [11,23]. In any case, thorough knowledge of normative development of DNA methylation levels is currently lacking and needed to interpret dissimilar estimates in the face of different measurement periods. Regarding the interpretation of the top hit, we further highlight that while stringent significance thresholds were used to reduce the risk of false positives, our current results may still reflect a chance finding and will need to be replicated in future studies.

To facilitate harmonization of the bullying exposure measurements in both cohorts, bullying exposure was defined with a lenient threshold. This implies that the difference in DNA methylation found for the CpG in *RAB14* is associated with exposure to bullying that is prevalent for children in the normal population. A more stringent definition of bullying might have brought forward different results, but a larger sample would be preferential for such an analysis. Additionally, with the current design we were unable to control for bullying exposure that participants might have been subjected to outside of the moment of measurement. More measurements of bullying exposure would likely lead to more precise estimates. Further, more questions on the different types of bullying in the Generation R Study would have permitted us to differentiate between specific bullying exposures. Multiple reporters of bullying would have been preferable as well, especially the current use of mother report in one cohort and child report in the other is suboptimal. For the *RAB14* CpG site, there was converging agreement however. Another constraint of the study was that the current selected samples were more affluent than the fuller populations of their respective cohorts, where ideally the full spectrum of characteristics for the children in our cohorts would be represented. Last, we do not know if changes in DNA methylation are the consequence of bullying exposure, or that such changes are associated with children who are more at risk of being bullied [50]. An experimental set-up, for example with an anti-bullying intervention [51], would shed more light on this.

In conclusion, the current study is the first to report an epigenome-wide hit related to bullying exposure. This CpG site is located in the *RAB14* gene and suggests that exposure bullying might be associated with Golgi apparatus functioning. The effect size was small, but in line with other population-based studies. Further, we found an enrichment for CpGs related to cardiac functioning and neurodevelopment, as well as for CpGs with low levels of methylation and sites for which DNA methylation decreased in exposed but increased in non-exposed. We believe that experimental and longitudinal research into DNA methylation is the path to a broader understanding of social stress and its effect on biological pathways.

## Methods

### Setting

Data were drawn from two population-based prospective birth cohorts, the Dutch Generation R Study (Generation R) and the British Avon Longitudinal Study of Parents and Children (ALSPAC). Pregnant women residing in the municipality of Rotterdam, the Netherlands, with an expected delivery date between April 2002 and January 2006 were invited to enrol in the Generation R Study. A more extensive description of the study can be found elsewhere [52]. The Generation R Study is conducted in accordance with the World Medical Association Declaration of Helsinki and has been approved by the Medical Ethics Committee of the Erasmus Medical Centre, Rotterdam. Written informed consent was obtained for all participants.

Pregnant women residing in the study area of former county Avon, United Kingdom, with an expected delivery date between April 1991 and December 1992 were invited to enrol in the ALSPAC study. Detailed information on the study design has been published previously [53,54]. The ALSPAC website contains details of all available data through a fully searchable data dictionary and variable search tool (http://www.bristol.ac.uk/alspac/researchers/our-data/). Ethical approval for the study was obtained from the ALSPAC Ethics and Law Committee and the Local Research Ethics Committees. Consent for biological samples has been collected in accordance with the Human Tissue Act (2004). Informed consent for the use of data collected via questionnaires and clinics was obtained from participants following the recommendations of the ALSPAC Ethics and Law Committee at the time.

In both cohorts DNA methylation was studied before and after reported bullying exposure. A timeline can be found in Supplemental Figure 1.

### Study population

In the Generation R Study, 9,778 pregnant mothers gave birth to 9,749 live-born children. For a subsample of 608 singletons DNA methylation data was collected at 6 and/or 10 years old (343 at both time points). Of these, 506 children had information available on bullying exposure and relevant covariates, including 289 children with DNA methylation available for both time points (Supplemental Figure 2(a)). This subsample consisted of participants with parents born in the Netherlands, with European ancestry confirmed based on genetic principle component analysis for all children with genetic data available (99.6% of the current sample).

In ALSPAC, the inclusion of 14,541 pregnant mothers resulted in 14,062 live births. DNA methylation was available at 7 and/or 17 years old for a subsample of 936 European singletons (877 at both time points) as part of the Accessible Resource for Integrated Epigenomic Studies (ARIES) study [55]. For 846 of these children data on bullying exposure and relevant covariates was available, including 793 children with DNA methylation data at both time points (Supplemental Figure 2(b)), leading to a combined sample size of 1,352 children in the meta-analysis. In each cohort, bullying exposure and covariates were compared between the selected sample and (i) a set of participants with complete data on covariates, irrespective of availability of data on bullying exposure or DNA methylation (*n *= 8,528 in Generation R and *n *= 12,393 in ALSPAC), and (ii) a set of participants with complete data on both covariates and bullying exposure, irrespective of availability of DNA methylation data (*n *= 4,336 in Generation R and *n *= 6,347 in ALSPAC).

### Bullying exposure

In Generation R, mothers filled out a questionnaire (adapted [56]) containing three questions on bullying exposure in the past few months, covering physical (‘In the past few months, how often has your child been bullied by way of spitting, hitting, kicking, or pinching?’), verbal (‘In the past few months, how often has your child been bullied by insulting, calling names or laughed at?’), and relational bullying (‘In the past few months, how often has your child been bullied by being excluded from activities, ignored by other children, or gossiped about?’). Items were rated on a 5-point scale (ranging from *never* to *several times a week*). In ALSPAC, bullying exposure was measured through self-report with an adapted version of the Bullying and Friendship Interview Schedule (BFIS) [57]. Nine questions covered physical (being hit or beaten up/belongings taken), verbal (threatened or blackmailed/tricked/called nasty names), and relational forms of bullying (others would not play with them/being made to do things they did not want to do/others told lies or nasty things about them/had games spoilt) in the preceding six months on a 4-point scale (ranging from *never* to *at least once a week*). Internal reliability of both measures was acceptable (Generation R: α = 0.74, ALSPAC: α = 0.73). Scores were dichotomized to harmonize the two bullying scales and avoid issues arising from extreme skewness of the data. Children were classified as being exposed to bullying if they were bullied ‘*at least once or twice in the past few months*’ on at least one of the items in Generation R, and at least ‘*1–3 times in the past six months*’ in ALSPAC [58–61].

### Variables sensitivity analyses

A more *stringent bullying exposure* variable was defined, in which children were classified as exposed when at least indicated to be bullied ‘*2 or 3 times a month’* in Generation R, and they at least indicated to be bullied ‘*about once a week’* in ALSPAC.

*Body Mass Index* (BMI) (kg/m2) was measured at 6 and 10 years in the Generation R and 7 and 17 years in the ALSPAC. Values were standardized to SD scores, adjusted for age and sex.

*Child behavioural problems* were measured at age 3 years with the mother-reported Child Behaviour Checklist for toddlers (CBCL1½-5) [62] in Generation R. Ninety-nine items were scored one a 3-point scale (range 0–2), regarding symptoms of anxiety, sadness, withdrawn behaviour, attention problems, and aggressive behaviours (α = 0.92). Items were summed into a weighed total problem behaviour scale, with 25% missing allowed. In ALSPAC, the mother-reported Strengths and Difficulties Questionnaire (SDQ) [63] at 4 years was used. The scales for emotional, conduct, and hyperactivity problems were used as a total problem behaviour score, consisting of 15 items, each rated on a 3-point scale (range 0–2) (α = 0.74). The remaining problem scale of the SDQ, the ‘peer problems’ scale, was excluded from the total score due to content overlap with bullying exposure.

*Child non-verbal intelligence quotient* (IQ) was measured by testing visuospatial abilities (Mosaics) and abstract reasoning (Categories) with the Snijders-Oomen Niet-verbale Intelligentie Test-Revisie (SON-R 2½-7) [64] at age 6 years in Generation R. In ALSPAC, a shortened version of the Wechsler Intelligence Scale for Children (3^rd^ UK edition (WISC-III)) [65] was measured at age 9 years.

O*ther stressful experiences* were measured in Generation R with a major life events inventory [66], reported by the mother, when the child was 10 years. This inventory covers stressful life events spanning the lifetime of the child, such as physical abuse, sexual abuse, conflict in the household, illness or death in the family, and parental separation. Three items related to bullying exposure were excluded, leaving 21 items (range 0–1). In ALSPAC, we used an Adverse Child Experiences (ACE) lifetime composite score [15,67]. This score is based on 541 questions mapping on to 10 ACEs up to age 16 years. Participants were included if there was at least 50% of the data available for each ACE. We excluded the ACE for bullying, leaving physical, sexual, and emotional abuse, emotional neglect, substance use in the household, violence between parents, parental mental health, parent conflict, parent offence, and parental separation (each range 0–1).

Last, a*lcohol use* was measured in ALSPAC with Alcohol Use Disorders Identification Test (AUDIT) at age 17. This tests consists of 10 items (range 0–4); a total score of 8 or more is considered hazardous [68].

### DNA methylation

Both cohorts used the EZ-96 DNA Methylation kit (Shallow) (Zymo Research Corporation, Irvine, USA) for bisulphite conversion on the extracted DNA. DNA methylation profiles were generated using the Illumina Infinium HumanMethylation450 BeadChip (Illumina Inc., San Diego, USA). Quality control and normalization steps can be found in Supplemental Methods. Analyses were restricted to 473,864 autosomal CpGs. DNA methylation levels are characterized by beta values (β values), representing the ratio of methylated signal relative to the sum of methylated and unmethylated signal measured per CpG. Outlying data points outside the 3*interquartile range were winsorized to the nearest point for each CpG. White blood cell (WBC) composition was estimated using the reference-based Houseman method [69]. Batch effects and additional unknown confounding were estimated using surrogate variable analysis (SVA) in *meffil* [70,71] in R version 3.4.3 [72].

### Statistical analyses

Associations between bullying exposure and changes in DNA methylation were analysed with a linear mixed model:

$$M_{ij}=\;\beta_{0}+\;u_{0i}+\;\beta_{1}Age_{ij}+\beta_{2}Bullied_{ij}+covariates\;+\epsilon_{ij}$$

Here, *M* denotes DNA methylation level, β_0_ fixed intercept, u_0i_ random intercept, β_1_ fixed age coefficient, β_2_ fixed bullying exposure coefficient, and *ϵ* random error. The *Bullied* variable was set to 0 for the first DNA methylation measurement and to 1 or 0 for the second measurement depending on whether the participant had been exposed to bullying, or not, respectively, and random intercept *u_0i_* allowed for inter-individual variation in DNA methylation at the first measurement. Participants are denoted by *i* and time points by *j*. Covariates included sex, gestational age, socio-economic status as indicated by highest attained educational level of the mother (low versus medium or high), surrogate variables (*n *= 20), WBCs (CD4 + T-lymphocytes, CD8 + T-lymphocytes, natural killer cells, B-lymphocytes, monocytes, and granulocytes). Current direct and second-hand smoking was adjusted for with the methylation level of *AHRR* cg05575921, which has proven to be a valid marker of tobacco exposure [73–76]. Methylation level for this CpG at both time points was entered into the equation, with levels divided into quintiles (as described elsewhere [77]) and lower levels indicating more smoking. Linear mixed models were applied using the *lme4* package [78].

To compare congruency between results from the two cohorts, estimates of the top 1000 CpGs in each cohort were correlated with estimates of those CpGs in the other cohort (as elsewhere [15]). Meta-analysis of estimates and standard errors of the two cohorts was performed using fixed models within the *metafor* [79] R package. To account for multiple testing (*n *= 473,864 CpGs), the significance threshold was set at a Bonferroni-corrected *p-*value of 1.06 × 10^−07^.

### Follow-up analyses

#### Sensitivity analyses

A series of sensitivity analyses were performed on CpGs with *p *< 1.06x10^−07^ in the meta-analysis. First, because the classification of bullying exposure is rather broad in the main analysis, we performed a sensitivity analysis with a more stringent dichotomization. Second, we reran analyses additionally adjusting for the following potential confounders or mediators that have been previously shown to associate with bullying exposure and DNA methylation. In each sensitivity analysis, one of the following variables was added to the main analysis: (i) Body Mass Index (BMI) (kg/m^2^) SD scores, adjusted for age and sex, at 6 and 10 years in Generation R and 7 and 17 years in ALSPAC (full sample size available) [60,80]; (ii) pre-existing behavioural problems (Generation R *n *= 451, ALSPAC *n *= 794) [5,50]; (iii) child non-verbal IQ (Generation R *n *= 465, ALSPAC *n *= 811) [81]; (iv) stressful experiences other than bullying exposure (Generation R *n *= 482, ALSPAC *n *= 597) [82,83]; and (v) alcohol use in ALSPAC (*n *= 624), where children are older [84,85]. For each sensitivity analysis, the coefficient for bullying exposure was compared with that for the main analysis with a z-test [86,87].

#### Genetic associations

DNA methylation for CpGs with *p *< 1.06x10^−07^ in the meta-analysis were tested for genetic associations in three ways. First, a look-up was performed in a list of CpGs located on a single nucleotide polymorphism (SNP), e.g. polymorphic CpGs [24]. Second, we tested for known associations with genetic variants, e.g. methylation quantitative trait loci, *in cis* (*cis* meQTLs) and *in trans* (*trans* meQTLs; http://www.mqtldb.org/; GCTA set) [25]. Third, we tested for additive genetic influences versus shared and unique environmental influences on the DNA methylation, as based on twin heritability analyses [26].

#### Look-up of previous findings in the literature

Previous studies on childhood adversity and DNA methylation, measured with the Illumina Infinium HumanMethylation450 BeadChip, were searched for current CpGs with *p *< 1.06x10^−07^ and their associated genes in the meta-analysis. Eleven studies were selected [11,13,15,16,27–32]. All studies examined childhood abuse or trauma, on study additionally examined bullying [13].

#### Candidate gene-wide analyses

A candidate gene follow-up analysis was conducted on the results stemming from the meta-analysis, for sites annotated to *5-HTT* and *NR3C1*. The significance threshold was set at a Bonferroni gene-level corrected *p-*value of 3.13 × 10^−03^ (*n *= 16 CpGs) and 1.22 × 10^−03^ (*n *= 41 CpGs), respectively.

#### Functional associations

Enrichment of Gene Ontology (GO) pathways was tested for genes associated with CpGs with *p *< 0.001 in the meta-analysis (cut-off described elsewhere [11]), while adjusting for gene size and pruning for redundant terms (a full method description can be found elsewhere [11]). Terms with *p *< 0.05 and more than one associated genes are reported, and highlighted if confirmed by near-identical terms from GO analyses with CpGs *p *< 0.01 and *p *< 0.001 in the meta-analysis.

## Supplementary Material

Supplemental Material

Supplemental Material

## References

[1] Craig W, Harel-Fisch Y, Fogel-Grinvald H, et al. A cross-national profile of bullying and victimization among adolescents in 40 countries. Int J Public Health. 2009;54:216–224. 19623475 10.1007/s00038-009-5413-9 PMC2747624

[2] Olweus D. Bullying at school. Aggressive behavior. Boston, MA: Springer; 1994. p. 97–130.

[3] Ringoot AP, Jansen PW, Rijlaarsdam J, et al. Self-reported problem behavior in young children with and without a DSM-disorder in the general population. Eur Psychiatry. 2017;40:110–115. 27992835 10.1016/j.eurpsy.2016.08.009

[4] Schoeler T, Duncan L, Cecil CM, et al. Quasi-experimental evidence on short-and long-term consequences of bullying victimization: A meta-analysis. Psychol Bull. 2018;144:1229. 30475016 10.1037/bul0000171

[5] Zwierzynska K, Wolke D, Lereya TS. Peer victimization in childhood and internalizing problems in adolescence: a prospective longitudinal study. J Abnorm Child Psychol. 2013;41:309–323. 22956274 10.1007/s10802-012-9678-8

[6] Copeland WE, Wolke D, Angold A, et al. Adult psychiatric outcomes of bullying and being bullied by peers in childhood and adolescence. JAMA Psychiatry. 2013;70:419–426. 23426798 10.1001/jamapsychiatry.2013.504 PMC3618584

[7] Winsper C, Lereya T, Zanarini M, et al. Involvement in bullying and suicide-related behavior at 11 years: a prospective birth cohort study. J Am Acad Child Adolesc Psychiatry. 2012;51:271–82. e3. 22365463 10.1016/j.jaac.2012.01.001

[8] Suderman M, Borghol N, Pappas JJ, et al. Childhood abuse is associated with methylation of multiple loci in adult DNA. BMC Med Genomics. 2014;7:13. 24618023 10.1186/1755-8794-7-13 PMC4007631

[9] Heijmans BT, Tobi EW, Stein AD, et al. Persistent epigenetic differences associated with prenatal exposure to famine in humans. Proc Nat Acad Sci. 2008;105:17046–17049. 18955703 10.1073/pnas.0806560105 PMC2579375

[10] Rijlaarsdam J, Pappa I, Walton E, et al. An epigenome-wide association meta-analysis of prenatal maternal stress in neonates: A model approach for replication. Epigenetics. 2016;11:140–149. 26889969 10.1080/15592294.2016.1145329 PMC4846102

[11] Cecil CAM, Smith RG, Walton E, et al. Epigenetic signatures of childhood abuse and neglect: implications for psychiatric vulnerability. J Psychiatr Res. 2016;83:184–194. 27643477 10.1016/j.jpsychires.2016.09.010

[12] Rusiecki JA, Byrne C, Galdzicki Z, et al. PTSD and DNA methylation in select immune function gene promoter regions: A repeated measures case-control study of U.S. military service members. Front Psychiatry. 2013;4:1–12. 23805108 10.3389/fpsyt.2013.00056 PMC3690381

[13] Marzi SJ, Sugden K, Arseneault L, et al. Analysis of DNA methylation in young people: limited evidence for an association between victimization stress and epigenetic variation in blood. Am J Psychiatry. 2018;175:517–529. 29325449 10.1176/appi.ajp.2017.17060693 PMC5988939

[14] Labonté B, Suderman M, Maussion G, et al. Genome-wide epigenetic regulation by early-life trauma. Arch Gen Psychiatry. 2012;69:722–731. 22752237 10.1001/archgenpsychiatry.2011.2287 PMC4991944

[15] Houtepen LC, Hardy R, Maddock J, et al. Childhood adversity and DNA methylation in two population-based cohorts. Transl Psychiatry. 2018;8:266. 30510187 10.1038/s41398-018-0307-3 PMC6277431

[16] Houtepen LC, Vinkers CH, Carrillo-Roa T, et al. Genome-wide DNA methylation levels and altered cortisol stress reactivity following childhood trauma in humans. Nat Commun. 2016;7:10. 10.1038/ncomms10967 PMC4802173 26997371

[17] Jovanova OS, Nedeljkovic I, Spieler D, et al. DNA methylation signatures of depressive symptoms in middle-aged and elderly persons: meta-analysis of multiethnic epigenome-wide studies. JAMA Psychiatry. 2018;75:949–959. 29998287 10.1001/jamapsychiatry.2018.1725 PMC6142917

[18] Mulder RH, Rijlaarsdam J, Luijk MPCM, et al. Methylation matters: FK506 binding protein 51 (FKBP5) methylation moderates the associations of FKBP5 genotype and resistant attachment with stress regulation. Dev Psychopathol. 2017;29:491–503. 28401840 10.1017/S095457941700013X PMC5426551

[19] van Dongen J, Zilhão NR, Sugden K, et al. Epigenome-wide association study of attention-deficit/hyperactivity disorder symptoms in adults. Biol Psychiatry. 2019;86:599–607. 10.1016/j.biopsych.2019.02.016 PMC6717697 31003786

[20] Ouellet-Morin I, Wong CC, Danese A, et al. Increased serotonin transporter gene (SERT) DNA methylation is associated with bullying victimization and blunted cortisol response to stress in childhood: a longitudinal study of discordant monozygotic twins. Psychol Med. 2013;43:1813–1823. 23217646 10.1017/S0033291712002784 PMC4231789

[21] Efstathopoulos P, Andersson F, Melas PA, et al. NR3C1 hypermethylation in depressed and bullied adolescents. Transl Psychiatry. 2018;8:121. 29921868 10.1038/s41398-018-0169-8 PMC6008402

[22] Bjornsson HT, Sigurdsson MI, Fallin MD, et al. Intra-individual change over time in DNA methylation with familial clustering. Jama. 2008;299:2877–2883. 18577732 10.1001/jama.299.24.2877 PMC2581898

[23] Yang BZ, Zhang H, Ge W, et al. Child abuse and epigenetic mechanisms of disease risk. Am J Prev Med. 2013;44:101–107. 23332324 10.1016/j.amepre.2012.10.012 PMC3758252

[24] Chen Y-A, Lemire M, Choufani S, et al. Discovery of cross-reactive probes and polymorphic CpGs in the illumina infinium humanmethylation450 microarray. Epigenetics. 2013;8:203–209. 23314698 10.4161/epi.23470 PMC3592906

[25] Gaunt TR, Shihab HA, Hemani G, et al. Systematic identification of genetic influences on methylation across the human life course. Genome Biol. 2016;17:61. 27036880 10.1186/s13059-016-0926-z PMC4818469

[26] Hannon E, Knox O, Sugden K, et al. Characterizing genetic and environmental influences on variable DNA methylation using monozygotic and dizygotic twins. PLoS Genet. 2018;14:e1007544. 30091980 10.1371/journal.pgen.1007544 PMC6084815

[27] Mehta D, Klengel T, Conneely KN, et al. Childhood maltreatment is associated with distinct genomic and epigenetic profiles in posttraumatic stress disorder. Proc Natl Acad Sci U S A. 2013;110:8302–8307. 23630272 10.1073/pnas.1217750110 PMC3657772

[28] Prados J, Stenz L, Courtet P, et al. Borderline personality disorder and childhood maltreatment: A genome-wide methylation analysis. Genes Brain Behav. 2015;14:177–188. 25612291 10.1111/gbb.12197

[29] Suderman M, Pappas JJ, Borghol N, et al. Lymphoblastoid cell lines reveal associations of adult DNA methylation with childhood and current adversity that are distinct from whole blood associations. Int J Epidemiol. 2015;44:1331–1340. 26351305 10.1093/ije/dyv168

[30] Kumsta R, Kreppner J, Kennedy M, et al. Psychological consequences of early global deprivation an overview of findings from the English & Romanian adoptees study. Eur Psychol. 2015;20:138–151.

[31] Marinova Z, Maercker A, Küffer A, et al. DNA methylation profiles of elderly individuals subjected to indentured childhood labor and trauma. BMC Med Genet. 2017;18:21. 28241754 10.1186/s12881-017-0370-2 PMC5329963

[32] Dunn EC, Soare TW, Zhu Y, et al. Sensitive periods for the effect of childhood adversity on DNA methylation: results from a prospective, longitudinal study. Biol Psychiatry. 2019;85:838–849. 30905381 10.1016/j.biopsych.2018.12.023 PMC6552666

[33] Lanner JT, Georgiou DK, Joshi AD, et al. Ryanodine receptors: structure, expression, molecular details, and function in calcium release. Cold Spring Harb Perspect Biol. 2010;2:a003996. 20961976 10.1101/cshperspect.a003996 PMC2964179

[34] Junutula JR, De Maziére AM, Peden AA, et al. Rab14 is involved in membrane trafficking between the Golgi complex and endosomes. Mol Biol Cell. 2004;15:2218–2229. 15004230 10.1091/mbc.E03-10-0777 PMC404017

[35] Biala Y, Bogoch Y, Bejar C, et al. Prenatal stress diminishes gender differences in behavior and in expression of hippocampal synaptic genes and proteins in rats. Hippocampus. 2011;21:1114–1125. 20623763 10.1002/hipo.20825

[36] Zhou J, Liu Z, Yu J, et al. Quantitative proteomic analysis reveals molecular adaptations in the hippocampal synaptic active zone of chronic mild stress-unsusceptible rats. Int J Neuropsychopharmacol. 2015;19:pyv100. 26364272 10.1093/ijnp/pyv100 PMC4772275

[37] Ornish D, Magbanua MJM, Weidner G, et al. Changes in prostate gene expression in men undergoing an intensive nutrition and lifestyle intervention. Proc Nat Acad Sci. 2008;105:8369–8374. 18559852 10.1073/pnas.0803080105 PMC2430265

[38] Sequeira A, Mamdani F, Ernst C, et al. Global brain gene expression analysis links glutamatergic and GABAergic alterations to suicide and major depression. PloS One. 2009;4:e6585. 19668376 10.1371/journal.pone.0006585 PMC2719799

[39] Das S, Boczan J, Gerwin C, et al. Regional and developmental regulation of syntaphilin expression in the brain: a candidate molecular element of synaptic functional differentiation. Mol Brain Res. 2003;116:38–49. 12941459 10.1016/s0169-328x(03)00212-2

[40] Shan J, Kushnir A, Betzenhauser MJ, et al. Phosphorylation of the ryanodine receptor mediates the cardiac fight or flight response in mice. J Clin Invest. 2010;120:4388–4398. 21099118 10.1172/JCI32726 PMC2993575

[41] Oltmann-Norden I, Galuska SP, Hildebrandt H, et al. Impact of the polysialyltransferases ST8SiaII and ST8SiaIV on polysialic acid synthesis during postnatal mouse brain development. J Biol Chem. 2008;283:1463–1471. 18045870 10.1074/jbc.M708463200

[42] Calandreau L, Márquez C, Bisaz R, et al. Differential impact of polysialyltransferase ST8SiaII and ST8SiaIV knockout on social interaction and aggression. Genes Brain Behav. 2010;9:958–967. 20659171 10.1111/j.1601-183X.2010.00635.x

[43] Park C, Lee S-A, Hong J-H, et al. Disrupted-in-schizophrenia 1 (DISC1) and Syntaphilin collaborate to modulate axonal mitochondrial anchoring. Mol Brain. 2016;9:69. 27370822 10.1186/s13041-016-0250-2 PMC4930613

[44] Sato C, Kitajima K. Impact of structural aberrancy of polysialic acid and its synthetic enzyme ST8SIA2 in schizophrenia. Front Cell Neurosci. 2013;7:61. 23675315 10.3389/fncel.2013.00061 PMC3646324

[45] Lin M-Y, Cheng X-T, Tammineni P, et al. Releasing syntaphilin removes stressed mitochondria from axons independent of mitophagy under pathophysiological conditions. Neuron. 2017;94:595–610. e6. 28472658 10.1016/j.neuron.2017.04.004 PMC5484086

[46] Mikkonen M, Soininen H, Tapiola T, et al. Hippocampal plasticity in Alzheimer’s disease: changes in highly polysialylated NCAM immunoreactivity in the hippocampal formation. Eur J Neurosci. 1999;11:1754–1764. 10215928 10.1046/j.1460-9568.1999.00593.x

[47] Panni T, Mehta AJ, Schwartz JD, et al. Genome-wide analysis of DNA methylation and fine particulate matter air pollution in three study populations: KORA F3, KORA F4, and the normative aging study. Environ Health Perspect. 2016;124:983–990. 26731791 10.1289/ehp.1509966 PMC4937859

[48] Green BB, Karagas MR, Punshon T, et al. Epigenome-wide assessment of DNA methylation in the placenta and arsenic exposure in the New Hampshire Birth Cohort Study (USA). Environ Health Perspect. 2016;124:1253–1260. 26771251 10.1289/ehp.1510437 PMC4977055

[49] Sharp GC, Arathimos R, Reese SE, et al. Maternal alcohol consumption and offspring DNA methylation: findings from six general population-based birth cohorts. Cohorts for H, Aging Research in Genomic Epidemiology plus methylation alcohol working g, et al. Epigenomics. 2018;10: 27–42. 29172695 10.2217/epi-2017-0095 PMC5753623

[50] Singham T, Viding E, Schoeler T, et al. Concurrent and longitudinal contribution of exposure to bullying in childhood to mental health: the role of vulnerability and resilience. JAMA Psychiatry. 2017;74:1112–1119. 28979965 10.1001/jamapsychiatry.2017.2678 PMC5710218

[51] Waasdorp TE, Bradshaw CP, Leaf PJ. The impact of schoolwide positive behavioral interventions and supports on bullying and peer rejection: A randomized controlled effectiveness trial. Arch Pediatr Adolesc Med. 2012;166:149–156. 22312173 10.1001/archpediatrics.2011.755

[52] Kooijman MN, Kruithof CJ, van Duijn CM, et al. The generation R Study: design and cohort update 2017. Eur J Epidemiol. 2016;31:1243–1264. 28070760 10.1007/s10654-016-0224-9 PMC5233749

[53] Boyd A, Golding J, Macleod J, et al. Cohort profile: the ‘children of the 90s’—the index offspring of the Avon Longitudinal Study of parents and children. Int J Epidemiol. 2013;42:111–127. 22507743 10.1093/ije/dys064 PMC3600618

[54] Fraser A, Macdonald-Wallis C, Tilling K, et al. Cohort profile: the Avon Longitudinal Study of Parents and Children: ALSPAC mothers cohort. Int J Epidemiol. 2012;42:97–110. 22507742 10.1093/ije/dys066 PMC3600619

[55] Relton CL, Gaunt T, McArdle W, et al. Data resource profile: accessible resource for integrated epigenomic studies (ARIES). Int J Epidemiol. 2015;44:1181–1190. 25991711 10.1093/ije/dyv072 PMC5593097

[56] Perren S, Alsaker FD. Social behavior and peer relationships of victims, bully‐victims, and bullies in kindergarten. J Child Psychol Psychiatry. 2006;47:45–57. 16405640 10.1111/j.1469-7610.2005.01445.x

[57] Wolke D, Woods S, Stanford K, et al. Bullying and victimization of primary school children in England and Germany: prevalence and school factors. Br J Psychol. 2001;92:673–696. 11762868 10.1348/000712601162419

[58] Horwood J, Waylen A, Herrick D, et al. Common visual defects and peer victimization in children. Invest Ophthalmol Vis Sci. 2005;46:1177–1181. 15790876 10.1167/iovs.04-0597

[59] Paget A, Parker C, Heron J, et al. Which children and young people are excluded from school? Findings from a large B ritish birth cohort study, the A von L ongitudinal S tudy of P arents and C hildren (ALSPAC). Child Care Health Dev. 2018;44:285–296. 28913834 10.1111/cch.12525

[60] Jansen PW, Verlinden M, Dommisse-van Berkel A, et al. Teacher and peer reports of overweight and bullying among young primary school children. Pediatrics. 2014;134:473–480. 10.1542/peds.2013-3274 25157018

[61] Verlinden M, Jansen PW, Veenstra R, et al. Preschool attention-deficit/hyperactivity and oppositional defiant problems as antecedents of school bullying. J Am Acad Child Adolesc Psychiatry. 2015;54:571–579. 26088662 10.1016/j.jaac.2015.05.002 PMC10424252

[62] Achenbach TM, Rescorla LA. Manual for the ASEBA preschool forms and profiles. Burlington, VT: University of Vermont, Research center for children, youth; 2000.

[63] Goodman R. The Strengths and Difficulties Questionnaire: a research note. J Child Psychol Psychiatry. 1997;38:581–586. 9255702 10.1111/j.1469-7610.1997.tb01545.x

[64] Tellegen PJ, Winkel M, Wijnbergen-Williams BJ. Snijders-Oomen Niet-verbale Intelligentietest-Revisie SON-R 2½–7. Lisse: Swets & Zeitlinger; 1996.

[65] Wechsler D. Wechsler Intelligence Scale for Children–3rd UK ed (WISC–III UK). Sidcup, UK: The Psychological Corporation; 1992.

[66] Dunn EC, Nishimi K, Neumann A, et al. Time-dependent effects of exposure to physical and sexual violence on psychopathology symptoms in late childhood: in search of sensitive periods in development. J Am Acad Child Adolesc Psychiatry. 2019;59:283–295. 10.1016/j.jaac.2019.02.022 PMC7448802 31078631

[67] Houtepen LC, Heron J, Suderman MJ, et al. Adverse childhood experiences in the children of the Avon Longitudinal Study of Parents and Children (ALSPAC). Wellcome Open Res. 2018;3:106. 10.12688/wellcomeopenres.14716.1 PMC6281007 30569020

[68] Saunders JB, Aasland OG, Babor TF, et al. Development of the alcohol use disorders identification test (AUDIT): WHO collaborative project on early detection of persons with harmful alcohol consumption‐II. Addiction. 1993;88:791–804. 8329970 10.1111/j.1360-0443.1993.tb02093.x

[69] Houseman EA, Accomando WP, Koestler DC, et al. DNA methylation arrays as surrogate measures of cell mixture distribution. BMC Bioinformatics. 2012;13:1. 22568884 10.1186/1471-2105-13-86 PMC3532182

[70] Leek JT, Johnson WE, Parker HS, et al. The sva package for removing batch effects and other unwanted variation in high-throughput experiments. Bioinformatics. 2012;28:882–883. 22257669 10.1093/bioinformatics/bts034 PMC3307112

[71] Leek JT, Storey JD. Capturing heterogeneity in gene expression studies by surrogate variable analysis. PLoS Genet. 2007;3:e161. 10.1371/journal.pgen.0030161 PMC1994707 17907809

[72] R Core Team. R: A language and environment for statistical computing, Vienna, Austria. 2013. https://www.R-project.org/.

[73] Zeilinger S, Kühnel B, Klopp N, et al. Tobacco smoking leads to extensive genome-wide changes in DNA methylation. PloS One. 2013;8:e63812. 23691101 10.1371/journal.pone.0063812 PMC3656907

[74] Philibert R, Hollenbeck N, Andersen E, et al. Reversion of AHRR demethylation is a quantitative biomarker of smoking cessation. Front Psychiatry. 2016;7:55. 27092088 10.3389/fpsyt.2016.00055 PMC4822186

[75] Reynolds LM, Magid HS, Chi GC, et al. Secondhand tobacco smoke exposure associations with DNA methylation of the aryl hydrocarbon receptor repressor. Nicotine Tob Res. 2016;19:442–451. 10.1093/ntr/ntw219 PMC6075517 27613907

[76] Dogan MV, Lei M-K, Beach SRH, et al. Alcohol and tobacco consumption alter hypothalamic pituitary adrenal axis DNA methylation. Psychoneuroendocrinology. 2016;66:176–184. 26821212 10.1016/j.psyneuen.2016.01.018 PMC4788520

[77] Bojesen SE, Timpson N, Relton C, et al. AHRR (cg05575921) hypomethylation marks smoking behaviour, morbidity and mortality. Thorax. 2017;72:646–653. 28100713 10.1136/thoraxjnl-2016-208789 PMC5520281

[78] Bates D, Mächler M, Bolker B, et al. Fitting linear mixed-effects models using lme4. arXiv Preprint arXiv:14065823. 2014.

[79] Viechtbauer W. Conducting meta-analyses in R with the metafor package. J Stat Softw. 2010;36:1-48.

[80] Fradin D, P-Y B, Belot M-P, et al. Genome-wide methylation analysis identifies specific epigenetic marks in severely obese children. Sci Rep. 2017;7:46311. 28387357 10.1038/srep46311 PMC5384222

[81] Verlinden M, Veenstra R, Ghassabian A, et al. Executive functioning and non-verbal intelligence as predictors of bullying in early elementary school. J Abnorm Child Psychol. 2014;42:953–966. 24337736 10.1007/s10802-013-9832-y

[82] Jansen PW, Verlinden M, Dommisse-van Berkel A, et al. Prevalence of bullying and victimization among children in early elementary school: do family and school neighbourhood socioeconomic status matter? BMC Public Health. 2012;12:494. 22747880 10.1186/1471-2458-12-494 PMC3575320

[83] Holt MK, Kaufman Kantor G, Finkelhor D. Parent/child concordance about bullying involvement and family characteristics related to bullying and peer victimization. J Sch Violence. 2008;8:42–63.

[84] Rospenda KM, Richman JA, Wolff JM, et al. Bullying victimization among college students: negative consequences for alcohol use. J Addict Dis. 2013;32:325–342. 24325767 10.1080/10550887.2013.849971 PMC3861792

[85] Zhang R, Miao Q, Wang C, et al. Genome‐wide DNA methylation analysis in alcohol dependence. Addict Biol. 2013;18:392–403. 23387924 10.1111/adb.12037

[86] Paternoster R, Brame R, Mazerolle P, et al. Using the correct statistical test for the equality of regression coefficients. Criminology. 1998;36:859–866.

[87] Clogg CC, Petkova E, Haritou A. Statistical methods for comparing regression coefficients between models. Am J Sociol. 1995;100:1261–1293.

