# Supplementary material for: Epigenomics of being bullied: changes in DNA methylation following bullying exposure
Source: Epigenetics. 2020 Jan 28;15(6-7):750–64. doi: 10.1080/15592294.2020.1719303 (PMC7574379; doi:10.1080/15592294.2020.1719303)
Supplement: Supplemental Material [file KEPI_A_1719303_SM9843.docx]

Supplemental Material - *Epigenomics of being bullied: Changes in DNA methylation following bullying exposure*

**Supplemental Methods**

*Quality control and normalization of DNA methylation*

In Generation R, quality control was performed using the CPACOR workflow^1^ on all 2,467 available DNA methylation samples, including cord blood samples (1,475 cord bloods, 500 peripheral bloods at 6 years, and 492 peripheral bloods at 10 years). Arrays with observed technical problems such as failed bisulfite conversion, hybridization or extension, as well as arrays with a mismatch between sex of the proband and sex determined by the chr X and Y probe intensities were removed from subsequent analyses. Additionally, only arrays with a call rate>95% per sample were processed further, resulting in 2,355 samples, 44 of which were sibling pairs. Hence 2,333 were carried forward into normalization.

In ALSPAC, quality control was performed on 6,057 samples (including 1,127 from cord blood, 1086 from peripheral blood at 7 years, and 1,073 from peripheral blood at 17 years from ALSPAC children and 2,771 peripheral bloods from their parents),using the *meffil* package^2^. After removing samples with mismatched genotypes, mismatched gender, incorrect relatedness, low concordance with samples collected at other time points, extreme dye bias, and poor probe detection, 5,337 samples remained, including 2,845 samples for 1,003 children.

To minimize cohort effects as much as possible, we normalized both cohorts together as a single dataset. In detail, functional normalization (10 control probe principal components, slide included as a random effect) was performed in R version 3.4.3^3^ with the *meffil* package on a combined Generation R and ALSPAC set including cord and whole blood samples comprising a total of 5,178 samples for a total of 485,512 CpG sites.

**Supplemental Analysis**

*Associations per time-point*

The association between bullying exposure and DNA methylation at cg17312179 was analyzed separately for each time-point (pre- (T1) and post-measurement (T2) of bullying exposure), to understand if reported change stemmed from a larger difference between exposed and non-exposed children after bullying report, rather than beforehand. Associations between bullying exposure at 6 years in Generation R and at 7 years in ALSPAC (combined *n*=1,224) were meta-analyzed, as were those at 10 years in Generation R and 17 years in ALSPAC (combined *n*=1,210). Results showed that the association with DNA methylation change at cg17312179 was driven by a difference in exposed versus non-exposed individuals at T2 (*b*=-2.43x10^-03^, SE=5.20x10^-04^, *p*=3.04x10^-06^), not at T1 (*b*=7.24x10^-04^, SE=4.86x10^-04^, *p*=1.37x10^-01^). This was confirmed by a z-test^4, 5^ between the coefficients for the two time-points (*p*=9.64x10^-06^).

**Supplemental Tables**

Supplemental Table 1. Missingness analysis: Comparison of sample characteristics across different selected sample sets in Generation R and ALSPAC

|  |  | **Generation R** | | | | |  | **ALSPAC** | | | | |
| --- | --- | --- | --- | --- | --- | --- | --- | --- | --- | --- | --- | --- |
|  |  | **Set 1** | **Set 2** | **Set 3** |  |  |  | **Set 1** | **Set 2** | **Set 3** |  |  |
|  |  | Participants with complete data on covariates^a^ | Participants in Set 1 and complete data on bullying exposure | Participants in Set 2 and complete data on DNA methylation | Set 1 versus  Set 3  *p*-value^b^ | Set 2 versus  Set 3  *p*-value^b^ |  | Participants with complete data on covariates^a^ | Participants in Set 1 and complete data on bullying exposure | Participants in Set 2 and complete data on DNA methylation | Set 1 versus  Set 3  *p*-value^b^ | Set 2 versus  Set 3  *p*-value^b^ |
| main analysis | *n* | 8528 | 4336 | 506 |  |  |  | 12393 | 6347 | 846 |  |  |
|  | Sex (No. (%) boys) | 4315 (50.6) | 2159 (49.8) | 251 (49.6) | 6.98e-01 | 9.75e-01 |  | 5996 (48.4) | 3189 (50.2) | 439 (51.9) | 5.24e-02 | 3.88e-01 |
|  | Gestational age in weeks (mean (SD)) | 39.7 (2.0) | 39.8 (1.8) | 40.2 (1.4) | **3.68e-12** | **3.95e-08** |  | 39.4 (1.9) | 39.5 (1.9) | 39.6 (1.5) | **5.66e-03** | **1.15e-02** |
|  | Maternal education (No. (%)) |  |  |  | **5.51e-36** | **8.87e-09** |  |  |  |  | **3.00e-27** | **2.15e-06** |
|  | Low | 2254 (26.4) | 644 (14.9) | 37 (7.3) |  |  |  | 2501 (20.2) | 815 (12.8) | 71 ( 8.4) |  |  |
|  | Medium | 2614 (30.7) | 1205 (27.8) | 112 (22.1) |  |  |  | 5509 (44.5) | 2751 (43.3) | 334 (39.5) |  |  |
|  | High | 3660 (42.9) | 2487 (57.4) | 357 (70.6) |  |  |  | 4383 (35.4) | 2781 (43.8) | 441 (52.1) |  |  |
|  | Maternal age at delivery (mean (SD)) | 30.48 (5.26) | 31.97 (4.52) | 32.80 (3.92) | **1.12e-32** | **1.17e-05** |  | 28.27 (4.86) | 29.23 (4.51) | 29.69 (4.35) | **8.45e-19** | **4.69e-03** |
|  | Age in years bullying exposure report | - | 8.07 (0.12) | 8.16 (0.23) | - | **1.15e-37** |  | - | 8.62 (0.27) | 8.56 (0.19) | - | **7.99e-14** |
|  | Bullying exposure (No. (%) yes) | - | 1828 (42.2) | 229 (45.3) | - | 1.98e-01 |  | - | 2500 (39.4) | 333 (39.4) | - | 1.00 |
| sensitivity analyses | *n* | - | 4336 | 506 |  |  |  | - | 6347 | 846 |  |  |
|  | Bullying exposure - sensitivity analysis (No. (%) yes) | - | 462 (10.7) | 50 (9.9) | - | 6.46e-01 |  | - | 1293 (20.4) | 174 (20.6) | - | 9.31e-01 |
|  | *n* | 6070 | 3926 | 404 |  |  |  | 7448 | 5802 | 820 |  |  |
|  | BMI (kg/m^2^) at T1 (mean (SD)) | 16.2 (1.9) | 16.0 (1.7) | 15.9 (1.3) | **1.11e-06** | **2.83e-02** |  | 16.2 (2.0) | 16.2 (2.0) | 16.2 (2.0) | 9.02e-01 | 6.67e-01 |
|  | *n* | 5230 | 3664 | 391 |  |  |  | 4573 | 3654 | 819 |  |  |
|  | BMI (kg/m^2^) at T2 (mean (SD)) | 17.5 (2.7) | 17.3 (2.5) | 17.1 (2.0) | **9.60e-06** | 5.52e-02 |  | 22.8 (4.2) | 22.7 (4.1) | 22.6 (3.6) | 9.43e-02 | 3.77e-01 |
|  | *n* | 4624 | 3399 | 451 |  |  |  | 9286 | 5695 | 794 |  |  |
|  | Behavioral problem score (mean (SD)) | 20.7 (15.1) | 19.8 (14.2) | 17.3 (12.2) | **5.03e-08** | **7.18e-05** |  | 6.9 (5.4) | 6.8 (4.8) | 6.9 (3.9) | 7.80e-01 | 9.67e-01 |
|  | *n* | 5558 | 3639 | 465 |  |  |  | 6339 | 5846 | 811 |  |  |
|  | Nonverbal intelligence quotient (mean (SD)) | 101.3 (15.1) | 103.5 (14.6) | 107.3 (14.0) | **7.57e-18** | **4.60e-08** |  | 100.0 (16.9) | 100.3 (16.8) | 102.6 (16.7) | **4.18e-05** | **2.62e-04** |
|  | *n* | 5045 | 3536 | 482 |  |  |  | 3646 | 3189 | 597 |  |  |
|  | Other stressful life events score (mean (SD)) | 4.1 (2.4) | 3.80 (2.2) | 3.7 (2.1) | **1.15e-04** | 1.63e-01 |  | 1.5 (1.4) | 1.5 (1.4) | 1.5 (1.4) | 4.05e-01 | 7.44e-01 |
|  | *n* | - | - | - |  |  |  | 3785 | 3105 | 624 |  |  |
|  | Alcohol use (mean (SD)) | - | - | - | **-** | **-** |  | 8.0 (4.8) | 8.1 (4.8) | 8.1 (4.8) | 4.78e-01 | 7.46e-01 |

SD: standard deviation; No.: number; T1: time point 1; T2: time point 2; BMI: Body Mass Index

^a^non-technical covariates, i.e.: child sex, gestational age, and education of the mother.

^b^*p*-value as based on T-test for numerical variables and Χ-square for categorical variables. Values in bold are *p*<0.05.

Supplemental Table 2. Functions of genes associated with the ten CpG sites with the lowest *p*-values in the meta-analysis of epigenome-wide associations with bullying exposure

| CpG site | Gene | Associated functions | |
| --- | --- | --- | --- |
| cg17312179 | *RAB14* | Golgi apparatus functioning, vesicle processing^6^ | |
| cg09291817 | *MAZ* | Inflammation induced, regulates amyloid A protein^7^ | |
| cg11278602 | *HCG4* | Unknown; located in major histocompatibility region, which is important for immune functioning^8^ | |
| cg00911813 | *TNRC18* | Unknown; other trinucleotide repeat (TNR) genes associated to transcription regulation and neurological disorders^9^ | |
| cg08971637 | *DGUOK* | Mitochondrial functioning^10^ | |
| cg12767834 | *SNPH* | Synaptic vesicle processing^11^ | |
| cg26394220 | *MIR375;*  *CCDC108* | Micro RNA, pancreas functioning^12^ | |
| cg19790568 | *PRX* | Peripheral myelin upkeep^13^ | |
| cg10929442 | *ST8SIA4* | Golgi apparatus functioning, neural cell plasticity^14^ | |
| ch.4.134822993R |  |  |  |

Supplemental Table 3. Meta-analyzed results for cg17312179 from main analysis and sensitivity analyses

|  |  |  | Added variable in sensitivity analysis^b^ | |  | Bullying exposure^c^ | |  | *Bullying exposure:*  main analysis  vs  sensitivity analysis^d^ |
| --- | --- | --- | --- | --- | --- | --- | --- | --- | --- |
|  |  | combined *n*^a^ | *B* (SE) | *p*-value |  | *B* (SE) | *p*-value |  | *p*-value |
| Main analysis | Bullying exposure | 1352 | - | - |  | -2.67e-03 (4.97e-04) | 7.17e-08 |  | - |
| Sensitivity analyses | Bullying exposure  (more stringent definition) | 1352 | - | - |  | -2.62e-03 (7.23e-04) | 2.95e-04 |  | 9.46e-01 |
|  | BMI | 1352 | -3.34e-05 (2.03e-04) | 8.69e-01 |  | -2.76e-03 (5.11e-04) | 6.62e-08 |  | 9.05e-01 |
|  | Behavioral problems | 1245 | -1.14e-05 (1.89e-05) | 5.44e-01 |  | -2.87e-03 (5.18e-04) | 2.92e-08 |  | 7.85e-01 |
|  | Intelligence quotient | 1276 | -7.82e-06 (1.28e-05) | 5.40e-01 |  | -2.64e-03 (5.18e-04) | 3.45e-07 |  | 9.61e-01 |
|  | Other stressful experiences | 1079 | 2.20e-04 (9.96e-05) | 2.73e-02 |  | -2.51e-03 (5.39e-04) | 3.18e-06 |  | 8.26e-01 |
|  | Alcohol use (only in ALSPAC) | 1130 | -1.09e-04 (7.33e-05) | 1.38e-01 |  | -2.91e-03 (5.23e-04) | 2.56e-08 |  | 7.42e-01 |

SE: standard error; BMI: Body Mass Index

^a^Sample size of Generation R + ALSPAC

^b^Each variable was added separately to the main analysis, while more stringent definition of bullying exposure replaced the bullying exposure variable in the main analysis. This was done in each cohort and estimates are based on the meta-analysis, except for alcohol, which was only available to ALSPAC.

^c^Based on meta-analysis

^d^Comparison performed with z-test on estimates of bullying exposure variable in main analysis versus bullying exposure variable in sensitivity analysis

**Supplemental Figures**

**
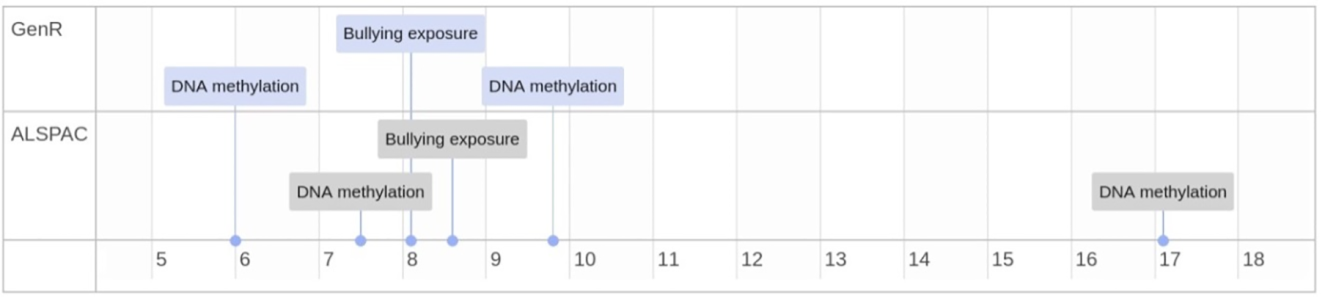
**

Supplemental Figure 1. Timeline of measurements in Generation R and ALSPAC


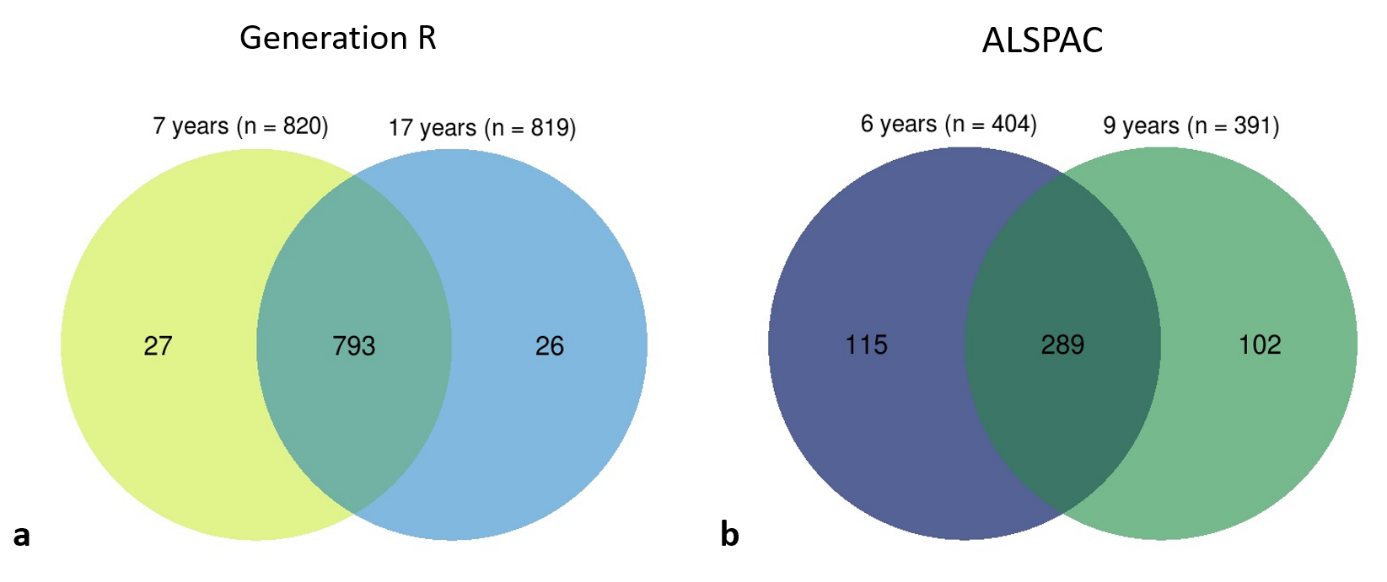


Supplemental Figure 2. Overlap samples sizes per time point (a) in Generation R (*n* data points=795, *n* participants=506) and (b) in ALSPAC (*n* data points=1,639, *n* participants=846)


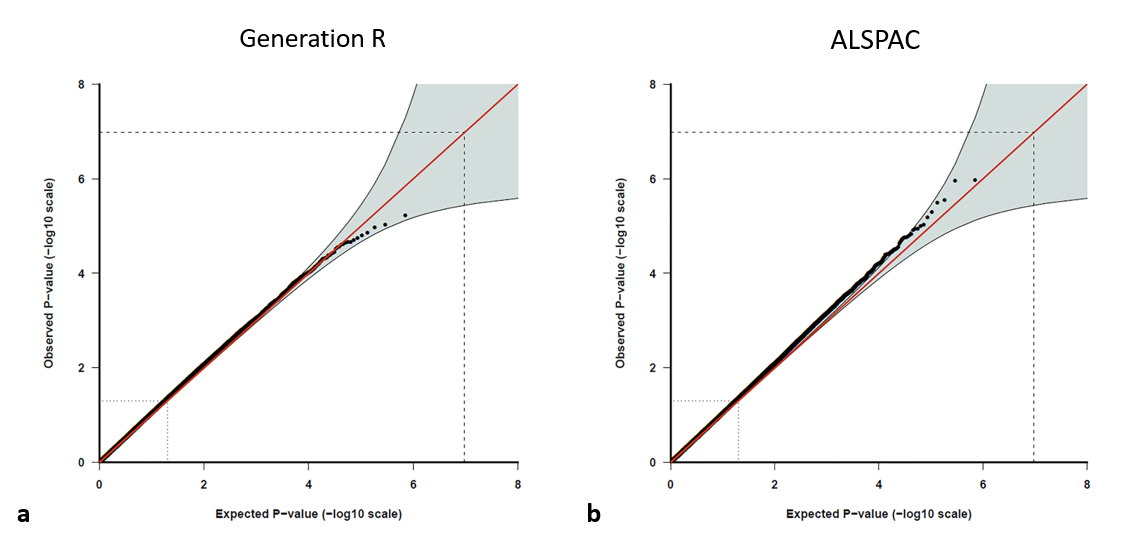


Supplemental Figure 3. QQ-plot of epigenome-wide study in (a) Generation R (λ=1.063) and (b) QQ- ALSPAC (λ=1.064)


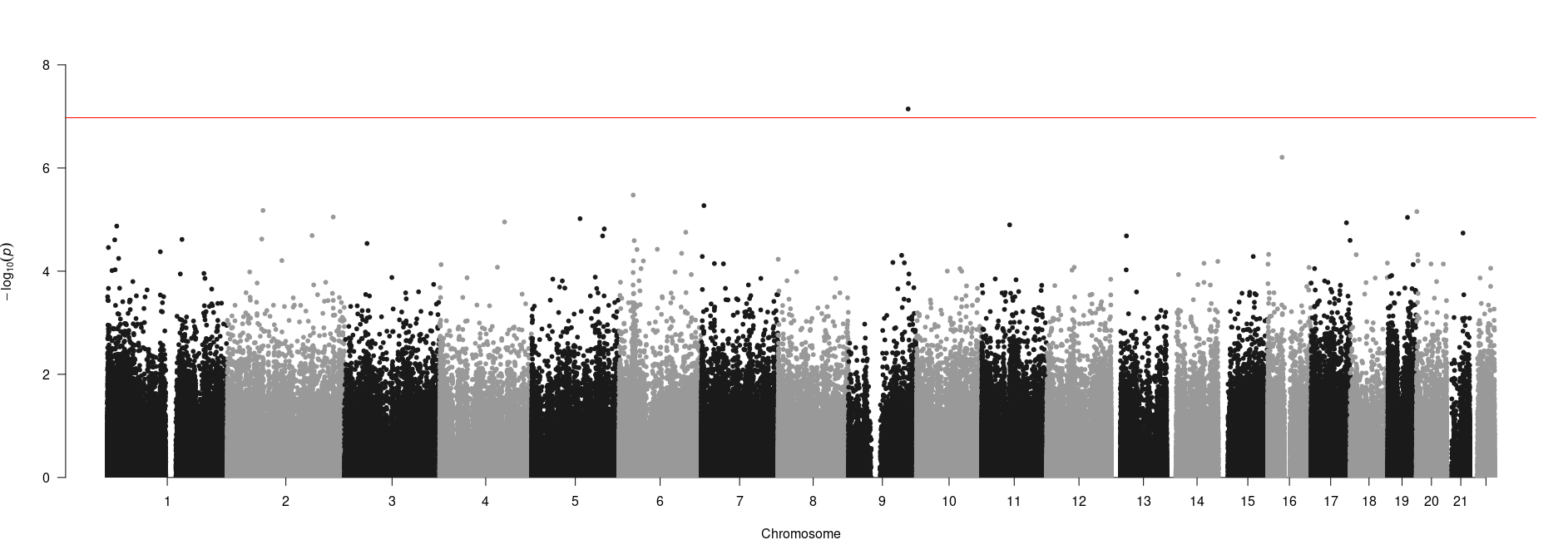


Supplemental Figure 4. Manhattan plot of -log10 *p*-values from meta-analysis


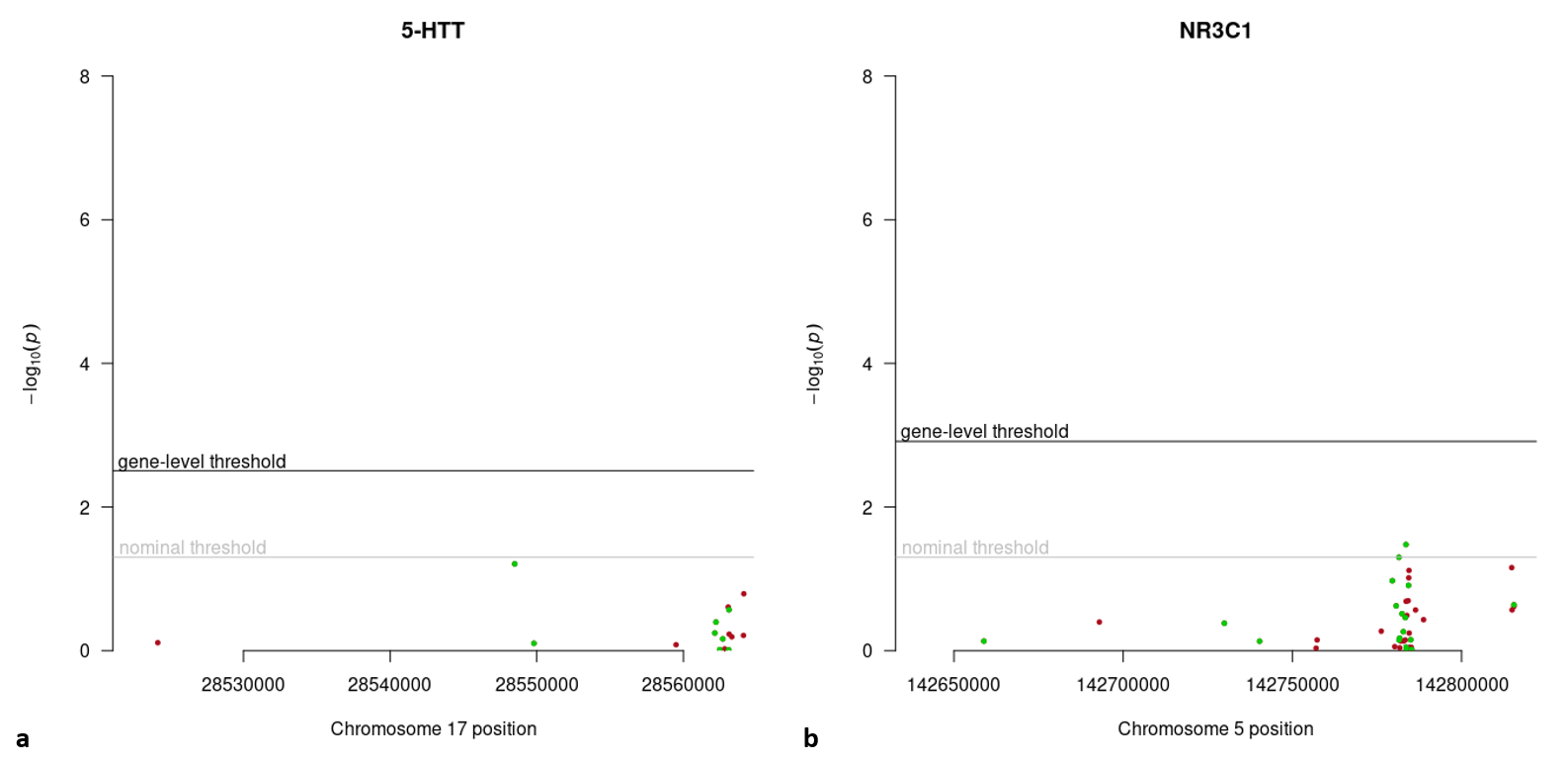


Supplemental Figure 5. Regional Manhattan plot of -log10 *p*-values from meta-analysis for candidate genes for the (a) serotonin transporter *5-HTT*, and (b) glucocorticoid receptor, *NR3C1*. Green points indicate a more positive change over time for the group exposed to bullying versus the non-exposed group, red points indicate a more negative change.


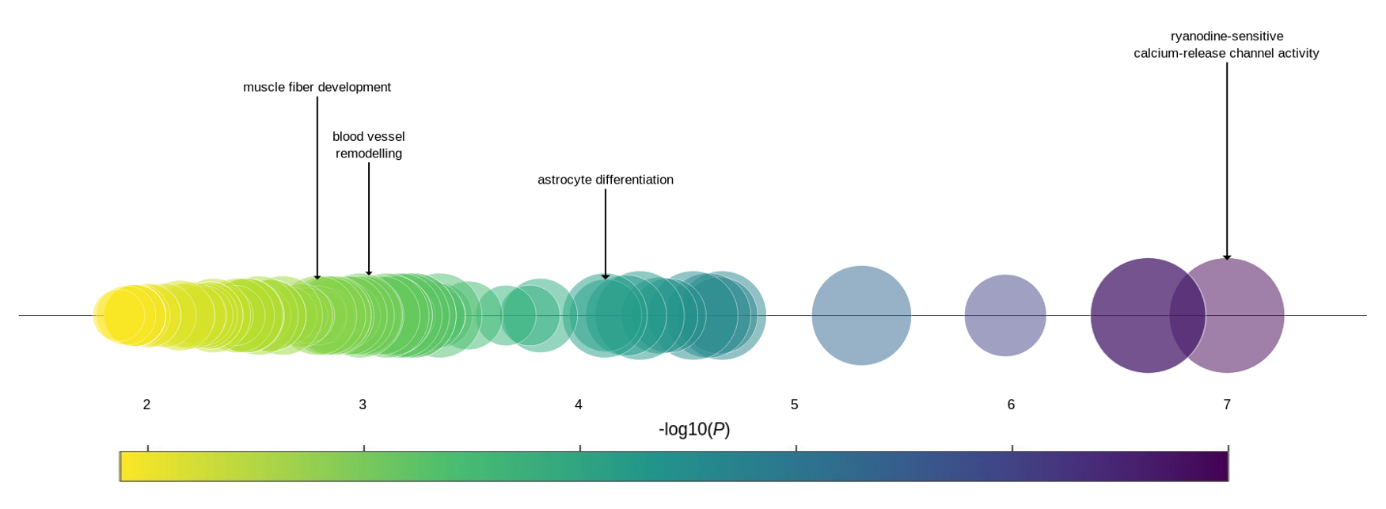


Supplemental Figure 6. Enriched biological processes (*p*<0.05) in Gene Ontology analysis of CpG sites with *p*<0.001 (*n=*644 CpG sites, *n*=396 genes) ordered by significance. Circle size represents percentage of genes represented in pathway versus all genes in that pathway.

**
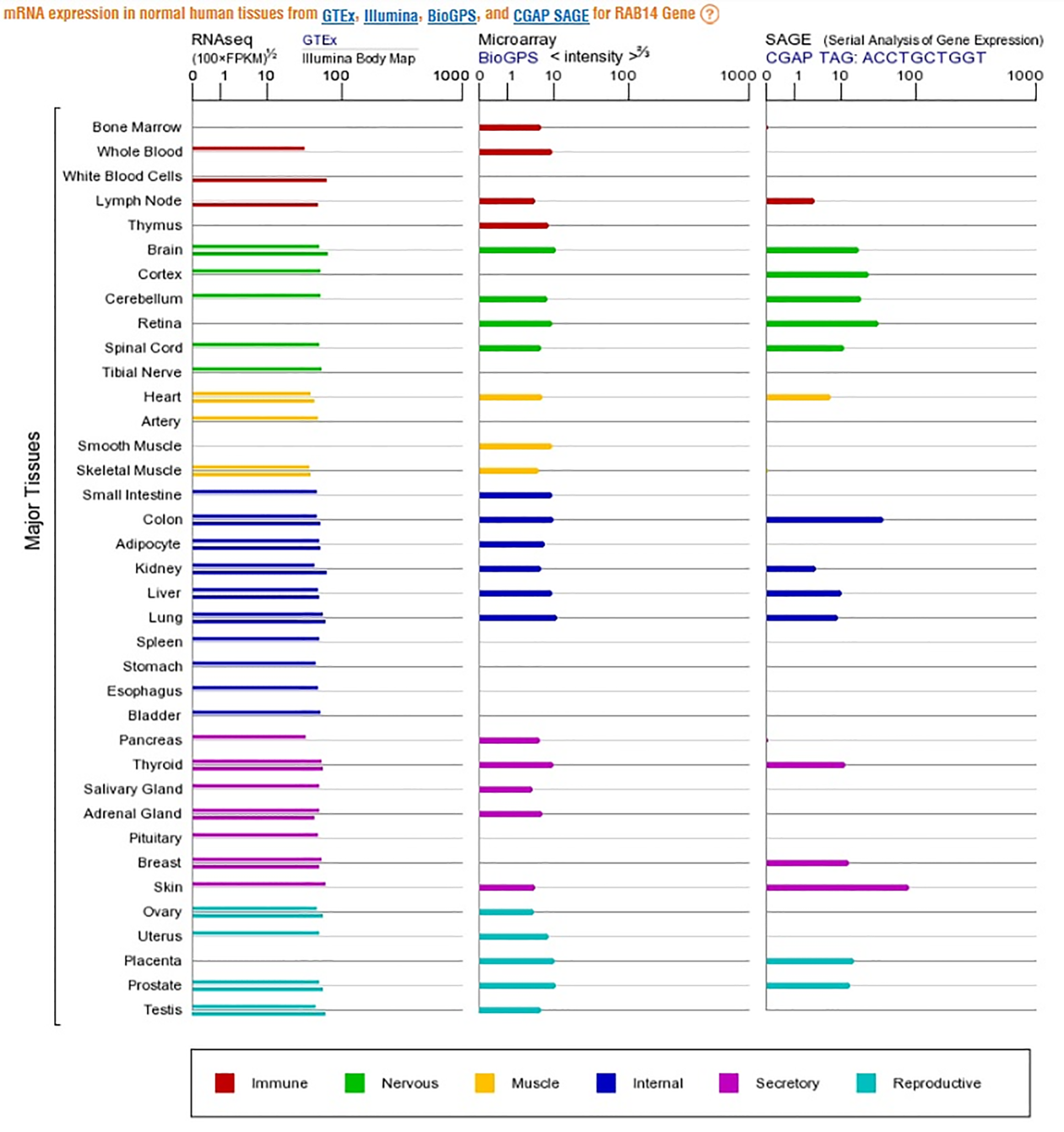
**

Supplemental Figure 7. *RAB14* mRNA expression in human tissues as portrayed by https://www.genecards.org/cgi-bin/carddisp.pl?gene=RAB14^15^

**References**

1. Lehne B, Drong AW, Loh M, Zhang W, Scott WR, Tan S-T, Afzal U, Scott J, Jarvelin M-R, Elliott P. A coherent approach for analysis of the Illumina HumanMethylation450 BeadChip improves data quality and performance in epigenome-wide association studies. Genome Biology 2015; 16:37.

2. Min JL, Hemani G, Davey Smith G, Relton C, Suderman M, Hancock J. Meffil: efficient normalization and analysis of very large DNA methylation datasets. Bioinformatics 2018.

3. R Core Team. R: A language and environment for statistical computing. 2013.

4. Paternoster R, Brame R, Mazerolle P, Piquero A. Using the correct statistical test for the equality of regression coefficients. Criminology 1998; 36:859-66.

5. Clogg CC, Petkova E, Haritou A. Statistical methods for comparing regression coefficients between models. American Journal of Sociology 1995; 100:1261-93.

6. Junutula JR, De Maziére AM, Peden AA, Ervin KE, Advani RJ, van Dijk SM, Klumperman J, Scheller RH. Rab14 is involved in membrane trafficking between the Golgi complex and endosomes. Molecular Biology of the Cell 2004; 15:2218-29.

7. Ray BK, Murphy R, Ray P, Ray A. SAF-2, a splice variant of SAF-1 acts as a negative regulator of transcription. Journal of Biological Chemistry 2002.

8. Shiina T, Inoko H, Kulski JK. An update of the HLA genomic region, locus information and disease associations: 2004. Tissue antigens 2004; 64:631-49.

9. Almeida B, Fernandes S, Abreu IA, Macedo-Ribeiro S. Trinucleotide repeats: a structural perspective. Frontiers in neurology 2013; 4:76.

10. Ronchi D, Garone C, Bordoni A, Gutierrez Rios P, Calvo SE, Ripolone M, Ranieri M, Rizzuti M, Villa L, Magri F. Next-generation sequencing reveals DGUOK mutations in adult patients with mitochondrial DNA multiple deletions. Brain 2012; 135:3404-15.

11. Das S, Boczan J, Gerwin C, Zald PB, Sheng Z-H. Regional and developmental regulation of syntaphilin expression in the brain: a candidate molecular element of synaptic functional differentiation. Molecular brain research 2003; 116:38-49.

12. Kloosterman WP, Lagendijk AK, Ketting RF, Moulton JD, Plasterk RHA. Targeted inhibition of miRNA maturation with morpholinos reveals a role for miR-375 in pancreatic islet development. PLoS biology 2007; 5:e203.

13. Gillespie CS, Sherman DL, Blair GE, Brophy PJ. Periaxin, a novel protein of myelinating Schwann cells with a possible role in axonal ensheathment. Neuron 1994; 12:497-508.

14. Oltmann-Norden I, Galuska SP, Hildebrandt H, Geyer R, Gerardy-Schahn R, Geyer H, Mühlenhoff M. Impact of the polysialyltransferases ST8SiaII and ST8SiaIV on polysialic acid synthesis during postnatal mouse brain development. Journal of Biological Chemistry 2008; 283:1463-71.

15. Stelzer G, Rosen N, Plaschkes I, Zimmerman S, Twik M, Fishilevich S, Stein TI, Nudel R, Lieder I, Mazor Y. The GeneCards suite: from gene data mining to disease genome sequence analyses. Current protocols in bioinformatics 2016; 54:1.30. 1-1.. 3.
